# Impact of Evening Light Exposures with Different Solid Angles on Circadian Melatonin Rhythms, Alertness, and Visual Comfort in an Automotive Setting

**DOI:** 10.3390/clockssleep4040047

**Published:** 2022-10-26

**Authors:** Michael Weng, Isabel Schöllhorn, Maryia Kazhura, Brian B. Cardini, Oliver Stefani

**Affiliations:** 1 Volkswagen AG, 38440 Wolfsburg, Germany; 2 Centre for Chronobiology, Psychiatric Hospital of the University of Basel, 4002 Basel, Switzerland; 3 Transfaculty Research Platform Molecular and Cognitive Neurosciences (MCN), University of Basel, 4001 Basel, Switzerland; 4 Department of Applied Psychology, University of Applied Sciences and Arts Northwestern Switzerland Basel, 4600 Olten, Switzerland

**Keywords:** bright light exposure, non-visual effects, non-image-forming, automotive interior lighting, solid angle, luminous surface size, subjective alertness, salivary melatonin, cognitive alertness

## Abstract

Future automotive interior lighting might have the potential to go beyond decorative purposes by influencing alertness, circadian physiology, and sleep. As the available space in the interior of an automobile for lighting applications is limited, understanding the impact of various luminous surface sizes on non-image-forming effects is fundamental in this field. In a laboratory study using a within-subject design, 18 participants were exposed to two bright light conditions with different solid angles and one dim light condition in a balanced, randomized order during the course of the evening. Our results demonstrate that both light conditions significantly increased subjective alertness and reduced salivary melatonin concentration but not cognitive performance compared to dim light. The solid angle of light exposure at constant corneal illuminance only affected visual comfort. While subjective alertness can be increased and melatonin can be attenuated with rather small luminaires, larger solid angles should be considered if visual comfort is a priority.

## 1. Introduction

Individually customizable ambient interior lighting has gained popularity in the automotive industry and is currently viewed as an important feature in upgrading car interiors. Such lighting fulfils a primarily decorative and practical purpose and facilitates the spatial perception of the car interior. Research, however, has shown that light not only enables humans’ visual perception but also has non-image-forming (NIF) effects. Light influences cognitive functions such as alertness, cognitive performance, and mood [1,2]. In addition, light contributes to the synchronization of circadian rhythmicity and helps humans to adjust their sleep–wake rhythm to the day–night cycle [3,4].

Besides rods and cones, there is a third photoreceptor type: the intrinsic photosensitive retinal ganglion cells (ipRGC), which contain the photopigment melanopsin, are distributed across the entire retina of the human eye [5,6], and are directly connected to the suprachiasmatic nuclei (i.e., the internal master clock [7,8]). Light exposure during the evening and in the night can suppress the release of melatonin—a hormone that is involved in the regulation and synchronization of the circadian rhythm and the increase in sleepiness [9,10,11,12,13]. Furthermore, exposure to blue- and white-appearing light with a high proportion of short-wave radiation during the evening or before sleep leads to longer sleep onset latencies and increased alertness levels [14,15]. These effects can be explained not only by the suppressing effect on melatonin but also by the direct influence of light on alertness via other neuronal pathways [16,17]. Thus, such light exposures might improve performance of shift workers, doctors, pilots, or drivers working during the night by counteracting fatigue [18]. While most of these observations were made under strict laboratory conditions, there are also some automotive field studies conducted in driving simulators [19,20,21,22,23] and under real driving conditions [24,25,26,27,28] with the goal to increase alertness and reduce fatigue by applying specific light exposures before or during driving. Due to the large differences in the characteristics of light exposures and inconsistent results within and between these studies, these investigations do not allow for clear conclusions to be drawn about the potential of appropriate automotive applications.

The solid angle of lighting exposure, which results from the illumination area and its distance to the illuminated person’s eye, could be a relevant factor to consider when designing biologically effective lighting modules for vehicle interiors [29]. Since ipRGCs are spread extensively across the retina [7,30], it can be assumed that non-visual effects of light are enhanced when light is emitted from a large surface area, as it would illuminate a greater area across the retina. Some studies indicate that larger solid angles of light exposure that keep the illuminance at the eye at a constant level might induce stronger melatonin suppressions than light exposures with a small luminous area and thus a smaller solid angle [31,32]. However, these studies either also varied the light color temperature [31] or lacked statistical significance, probably due to small sample size (*N* = 6) [32]. Recently, Niemeyer et al. (2020) investigated the influence of two morning light exposures with different solid angles (0.05sr vs. 0.44sr) at the eye on subjective alertness and cognitive performance [33]. They did not find significant differences for either of the two light exposures compared to dim light, which poses the question whether an increase in the solid angle can increase alertness and cognitive performance in the evening and at night.

The available space in the interior of an automobile for lighting applications is severely limited, and the distance between possible light units and the eyes of the passenger is generally smaller than in applications designed for buildings’ interiors. Therefore, it is of practical importance to investigate whether the size of the luminous area plays a role in NIF effects. In this work, we investigated the influences of two different solid angles of bright light exposure compared to dim light exposure in the evening on melatonin concentration, subjective alertness, and on the performance in two cognitive attention tasks. We expected an increase in melatonin due to its circadian nature, while we expected subjective and objective alertness to decrease according to circadian phase, time awake, and the occurrence of task-related fatigue [34,35]. We hypothesized that light exposure attenuates these increases and declines. Furthermore, we expected that a larger solid angle leads to a greater degree of melatonin suppression and a less pronounced decline in subjective alertness and cognitive performance.

## 2. Materials and Methods

### 2.1. Ethical Approval

The study protocol, questionnaires, and consent forms of the research project were reviewed and approved by the Ethics Committee Northwestern and Central Switzerland (EKNZ) for its compatibility with the valid national and international guidelines for research studies involving humans (BASEC reference number: 2019 00718). All participants gave written informed consent to the planned procedure of the study prior to their participation.

### 2.2. Study Participants

Healthy, male participants (20 to 40 years) were recruited for this study. Potential participants completed the following questionnaires: the Pittsburgh Sleep Quality Index (PSQI) [36], a German adaption of the Morningness–Eveningness Questionnaire (D-MEQ) [37], the SF-36 Health Status Questionnaire [38], as well as a short sociodemographic questionnaire. Based on the results of these questionnaires, 18 eligible male subjects aged 20 to 35 years (M = 25.5, SD = 3.92) were selected to participate in the experiment. Subjects with a good sleep quality (PSQI < 5) and no evening or extreme morning chronotypes (MEQ score: ≤30 and ≥42) were included in the study. Participants with a monocular visual acuity <0.7 (Freiburg Visual Acuity Test [39]) and with color vision deficiencies (<17 of 21 Ishihara plates [40]) were excluded from the study. Further exclusion criteria were drug consumption, medication, diagnosed mental illness, and cardiovascular or neurological disorders. Thus, according to G*Power 3.1.9.7 [41] and using the suggested study design, approximately medium to large effects (*η_p_*^2^ ≥ 0.089) can be detected with a power of 0.8 at an alpha level of 0.05. The subjects were asked to maintain a regular sleep–wake cycle over a period of three weeks starting 7 days prior to the study, which was documented in a sleep log. Subjects who completed the study were compensated with an allowance of 400 CHF.

### 2.3. Tasks and Measures

Saliva samples were scheduled every 30 min. Salivary melatonin levels were quantified using saliva melatonin kits (BÜHLMANN Laboratories AG, Schönenbuch, Switzerland) by a direct double-antibody radioimmunoassay (analytical least detectable dose: 0.3 pg/mL; Chrono@Work, Groningen, The Netherlands).

To assess subjective alertness, participants completed the Karolinska Sleepiness Scale (KSS) [42] at hourly intervals. In addition, a Visual Comfort Questionnaire (VCQ; also used by [43], derived from the results of [44]) was used at hourly intervals to assess the visual comfort of lighting based on pleasure, brightness, light color, glare, and the impact on alertness as well as concentration, each rated on a five-point scale.

Objective alertness was assessed at hourly intervals using a 10 min version of the Psychomotor Vigilance Task (PVT) [45] and a 20 min adaptation of the Mackworth Clock Test (MWCT) [46,47]. In the PVT, a white cross was displayed against a dark background and was replaced at regular intervals by a white stopwatch. The subjects’ task was to focus on the white cross and to react to an appearance of the stopwatch as quickly as possible by pressing a key on the keyboard. The interstimulus interval varied randomly between 2 and 10 s. Mean reaction times of each PVT session were evaluated to assess cognitive alertness (outliers ± 3 SD excluded). In the MWCT, subjects had to follow a white dot moving clockwise across 60 circles arranged in a circle. The white dot moved to the next circle at one-second intervals. At 12 random times (3 times in each 5 min segment, with a minimum interval of 8 s between two events), the dot skipped one of the circles, to which the subjects had to react as quickly as possible by pressing a key. A response was considered a miss if there was no reaction within 8 s after a skip event. The miss rate was considered as the performance measure for each MWCT session, defined as the quotient of the number of misses divided by the number of skips occurred.

### 2.4. Procedure

Each subject was invited to a controlled laboratory environment at the Centre for Chronobiology on three evenings (washout period between laboratory sessions was one week). Subjects were asked to spend as little time as possible in daylight and to refrain from consuming caffeinated beverages prior to the laboratory sessions. During the sessions, subjects underwent identical test procedures in each of the three lighting conditions (dim light, light exposure with small solid angle, light exposure with large solid angle) in pseudo-randomized order for 5 h. Subjects arrived in the laboratory 4.5 h before individual bed times. Within 90 min after arrival, subjects received instructions, were allowed to listen to an audio book, received a meal, and underwent a dark adaptation phase (<0.1lx) for the last 30 min prior to the start of the experiment. Subsequently, the light exposure started at 8:00 or 8:45 p.m., depending on individual bed times, and lasted 180 min. All measurements of the dependent variables are denoted in minutes relative to the start time of light exposure (e.g., *t*_30_ was collected 30 min after start of the light intervention). To ensure a constant gaze direction during the three-hour light exposure, participants had to watch a video of an autonomous drive, filmed from the driver’s perspective on a monitor, and respond to lane changes by pressing a key. At hourly intervals, this task was paused, and participants were asked to complete the 10 min PVT (*t*_30_, *t*_90_, *t*_150_) followed by the 20 min MWCT (*t*_40_, *t*_100_, *t*_160_) and the visual comfort questionnaire (*t*_60_, *t*_120_, *t*_180_). Starting 90 min before light exposure, subjects gave saliva samples to determine melatonin concentration at 30 min intervals (*t_-_*_90_–*t*_210_; *t_-_*_30_ was omitted because subjects consumed their meal immediately beforehand). Additionally, starting 60 min before light exposure, subjects completed the KSS at hourly intervals (*t_-_*_60_–*t*_180_).

### 2.5. Experimental Setup and Light Settings

**Figure 1:**
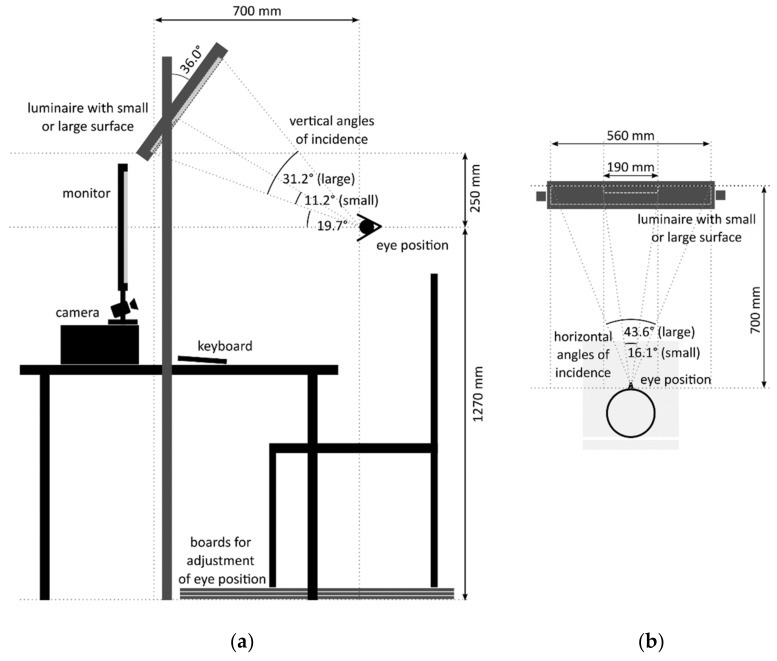
Side (**a**) and top view (**b**) of the experimental setup, illustrating the position of the subjects’ eyes and the angles of incidence. The angles refer to the two test conditions with a small (11.2° vertical, 16.1° horizontal) and large (31.2° vertical, 43.6° horizontal) solid angle.

**Figure 2:**
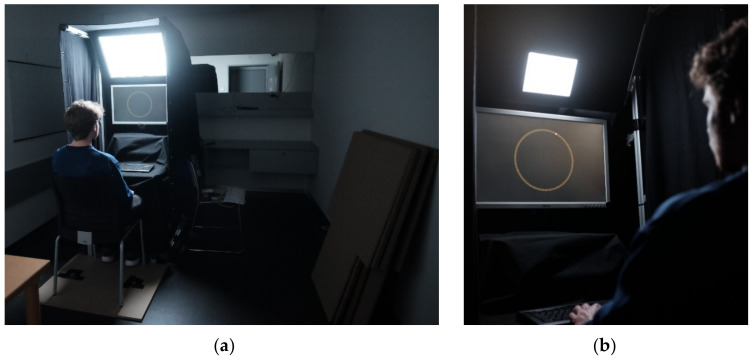
(**a**) Experimental setup in the test condition with the large solid angle; (**b**) subject completing the MWCT in the test condition with the small solid angle.

The design and spatial arrangement of the experimental luminaires are based on the study of Niemeyer et al. (2020) [33]. The experimental setup is illustrated schematically in Figure 1. Wooden boards were placed beneath the participant’s seat to adjust the individual vertical eye position relative to the floor to a height of 1270 mm. This ensured comparable lighting conditions at the eyes across all subjects. To minimize light reflections on the table and in the test room, the test setup was lined with black fabric. A camera and an audio communication system allowed the experimenter to observe the subjects during the experiment from a separate room and to communicate with the subjects in case they averted their gaze from the monitor or had any questions. Figure 2 illustrates the test setup and a participant completing the MWCT.

**Figure 3:**
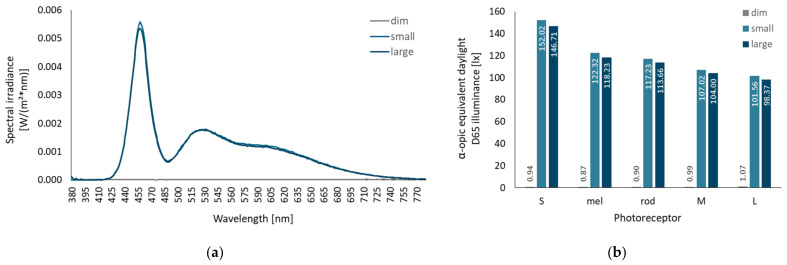
(**a**) Spectral irradiances vertical at eye position (see Figure 1a) used in the three experimental conditions; (**b**) equivalent daylight (D65) illuminances in the experimental conditions for the five photoreceptor S-cones, melanopsin, rods, M-cones, and L-cones according to the CIE S 026/E:2018 standard [48].

The experimental luminaire consists of 140 groups of six LEDs (one each of Osram DURIS^®^ GD PSLR31.13-2U-2-F-150 and GT PSLR31.13-MP-T1-E-150, two each of Osram SYNIOS^®^ KB DMLN31.13-5F-6-15 and KY DMLQ31.FY-7J-5F-8E-300). At a distance of 50 mm in front of the LED groups, an acrylic glass (WH02 GT) with dimensions of 560 × 400 mm was positioned as a diffuser to create a homogeneous light-emitting surface. In the test condition with the small solid angle, a masking black frame was placed in front of the acrylic glass, reducing the light-emitting surface to an area of 190 × 145 mm. The resulting solid angles were 0.393sr (small solid angle condition) and 0.051sr (large solid angle condition) at subjects’ eye position as shown in Figure 1. For each solid angle condition, luminaires were adjusted to produce a corneal illuminance of approximately 100lx and a Correlating Color Temperature (CCT) of ≥12,000 K, providing a melanopic equivalent daylight D65 illuminance (mEDI) of about 120lx (using LED-Meter MK350S, UPRTek, Jhunan, Taiwan). Control measurements resulted in an illuminance of 101.98lx (mEDI = 122.3lx) and 98.94lx (mEDI = 118.2lx) at the defined eye position in the experimental conditions with a small and large solid angle, respectively (mEDI calculated with the CIE S 026/E:2018 Toolbox [48]). In the dim light condition, the luminaire was not activated, so the screen was the only light source in the room. Control measurements revealed a corneal illuminance of 1.06lx and 0.87lx mEDI. Figure 3a illustrates the spectral irradiance vertical at eye position used in the three experimental conditions. Irradiances weighted for all photoreceptors are presented in Figure 3b. Table A1 in Appendix A provides an overview of all relevant photometric metrics.

### 2.6. Statistical Analysis

For each dependent variable, an analysis of variance (R package “afex” [49]) was performed with the two repeated-measures factors, “experimental condition” ((dim) light, (small) solid angle, (large) solid angle) and “time of measurement” (includes all times (*t_x_*) after the start of light exposure). To minimize the influence of inter- and intraindividual differences in the initial levels, KSS and melatonin values were analyzed as the difference to the values at *t*_0_ on the same experimental day, indicating the in- (positive values) or decrease (negative values) in the melatonin and alertness level, respectively, since the start of light exposure. The Mauchly test was used to test sphericity assumptions, and Greenhouse–Geisser epsilon-adjusted degrees of freedom and probabilities were reported in case of violations [50,51,52]. Where an analysis of variance revealed a significant main effect in the factor experimental condition, a contrast analysis was performed with respect to the research questions. To test the general effect of light exposure, the experimental condition dim was contrasted against the combination of the experimental conditions small and large. With another planned contrast, the experimental condition small was contrasted with the experimental condition large to determine the effect of the solid angle between the two conditions with light exposure. Differences were assumed to be significant at *p* < 0.05.

## 3. Results

### 3.1. Salivary Melatonin

**Figure 4:**
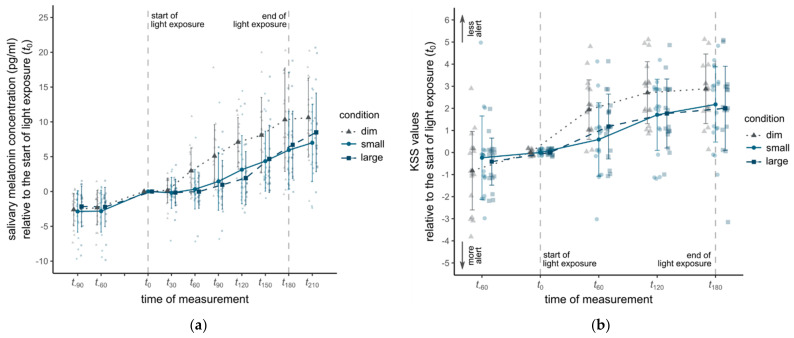
Mean values of salivary melatonin concentrations (**a**) and KSS scores (**b**) relative to the beginning of light exposure (*t*_0_). The error bars represent the standard deviations. The individual values are highlighted in pale colors.

**Figure A1:**
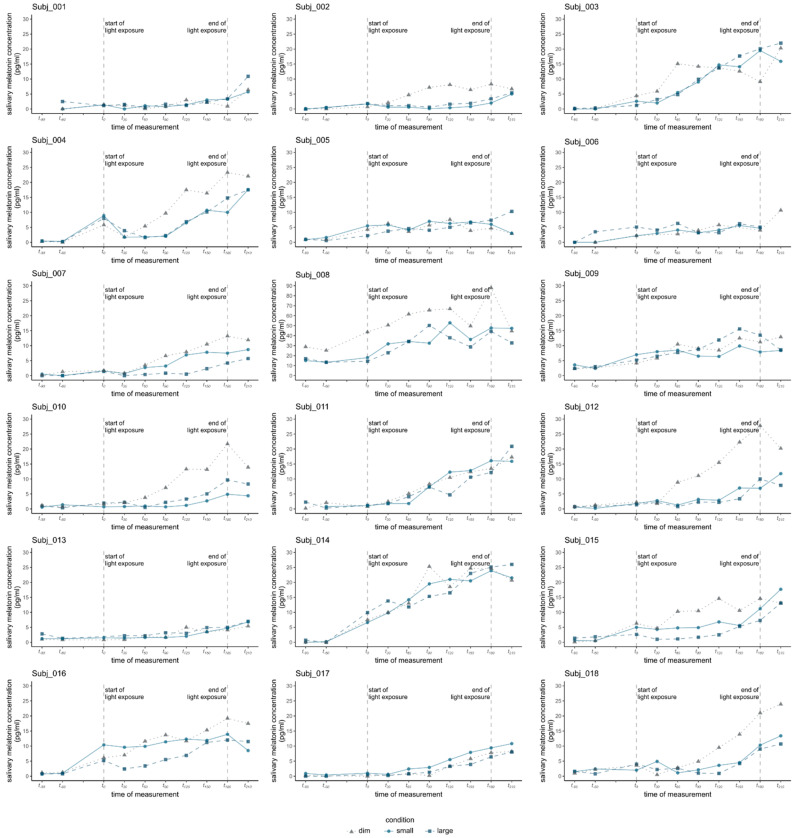
Individual courses of melatonin concentrations. Note: For subject Subj_008 a different scaling of y-axis is used. Due to the significant higher concentrations, subject Subj_008 was excluded from all other evaluations and visualizations.

The time course of the salivary melatonin concentrations is shown in Figure 4a. Melatonin concentration followed the typical evening profile with increasing levels during the laboratory sessions (F_1.92, 30.78_ = 38.99, *p* < 0.001, *η*^2^ = 0.34). In addition, the analysis yielded a significant main effect of experimental condition on melatonin levels indicating an effect of the light exposures on melatonin release (F_2, 32_ = 10.12, *p* < 0.001, *η*^2^ = 0.11). However, the interaction between the factors experimental condition and time of measurement was non-significant (F_3.11, 49.72_ = 2.63, *p* = 0.06). A planned contrast revealed lower melatonin concentrations in the two experimental conditions with light exposure (M = 3.19, SD = 5.02) compared to the dim condition (M = 6.33, SD = 5.91), t_16_ = 3.69, *p* = 0.002. Between the experimental conditions with a small (M = 3.14, SD = 4.99) and large solid angle (M = 3.23, SD = 5.07), melatonin concentration did not significantly differ (t_16_ = 0.15, *p* = 0.89). Individual melatonin profiles revealed large individual differences (see Appendix B Figure A1). Of the 18 subjects, 4 did not show a significant melatonin increase even during the dim light condition (≤5.0 pg/mL at *t*_180_). In addition, the expected melatonin suppression induced by each of the light exposure conditions could not be observed in four subjects who showed an increase in melatonin during the dim light condition (difference between dim and both light conditions at *t*_180_ ≤ 5.0 pg/mL).

### 3.2. Subjective Alertness

Figure 4b shows the time course of the KSS values in the three experimental conditions. As expected, KSS values increased over time (F_1.38, 22.07_ = 20.72, *p* < 0.001, *η*^2^ = 0.09) and differed significantly between the experimental conditions (F_2, 32_ = 3.48, *p* = 0.043, *η*^2^ = 0.08). The interaction between experimental condition and time of measurement was non-significant (F_4, 64_ = 1.38, *p* = 0.25). Contrast analysis revealed a statistically significant difference between the dim condition (M = 2.51, SD = 1.47) and the two experimental conditions with a small and large solid angle (M = 1.57, SD = 1.7) (t_16_ = 2.45, *p* = 0.026), confirming an alertness-enhancing effect of light exposure. However, mean KSS values in the light exposure conditions with a small (M = 1.49, SD = 1.76) and large solid angle (M = 1.65, SD = 1.66) did not differ significantly (t_16_ = 0.41, *p* = 0.69).

### 3.3. Cognitive Alertness

**Figure 5:**
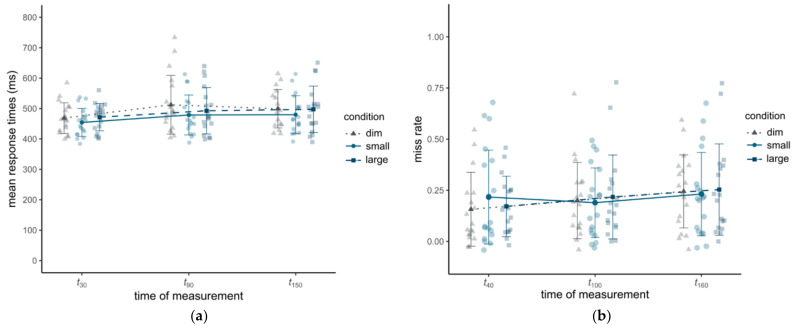
Mean values of reaction times in the PVT (**a**) and miss rates in the MWCT (**b**) for each time of measurement and experimental condition. The error bars represent the standard deviations. The individual mean values are pale shaded.

Mean reaction times in the PVT increased during all conditions, indicating a significant effect of time of measurement (see Figure 5a) (F_1.26, 20.11_ = 11.87, *p* < 0.001, *η^2^* = 0.04). However, there was no significant influence of experimental condition (F_2, 32_ = 1.33, *p* = 0.28) and no significant interaction effect between experimental condition and time of measurement (F_2.37, 37.86_ = 0.73, *p* = 0.51). An identical pattern can be seen for the miss rates in the MWCT (see Figure 5b). Miss rates increased over time (F_2, 34_ = 6.16, *p* = 0.005, *η*^2^ = 0.02). There was no effect of condition (F_2, 34_ = 0.09, *p* = 0.92) and no significant interaction (F_2.65, 45_ = 0.65, *p* = 0.57).

### 3.4. Visual Comfort

**Figure A2:**
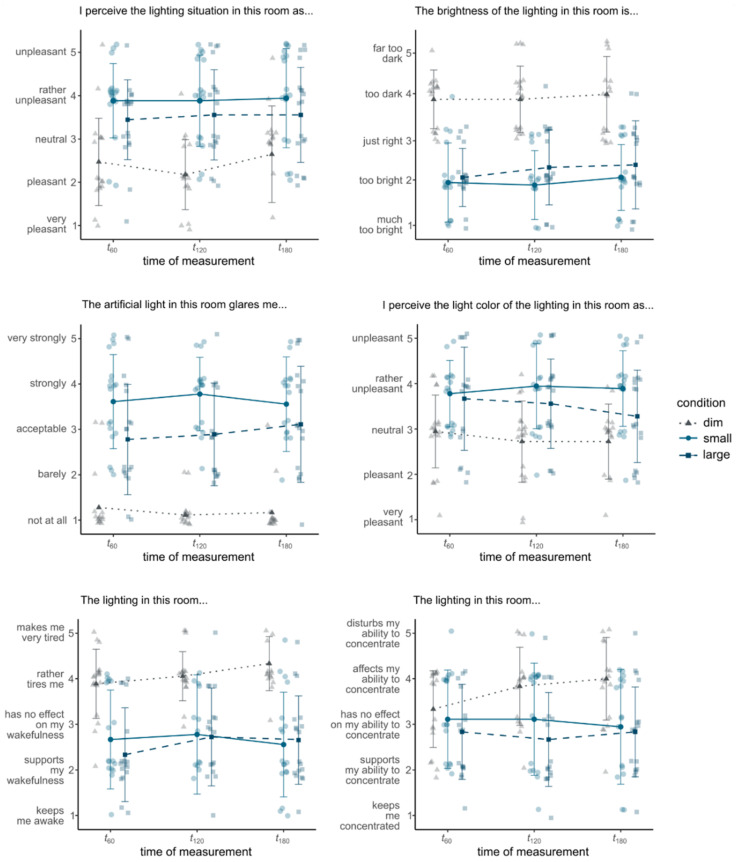
Mean values of the ratings of the six scales of the VCQ. Statistical analysis reveals a significant main effect of condition for lighting situation, F_1.40, 22.38_ = 15.08, *p* < 0.001, *η*^2^ = 0.29, brightness, F_2, 34_ = 50.25, *p* < 0.001, *η*^2^ = 0.54, glaring, F_2, 34_ = 62.78, *p* < 0.001, *η*^2^ = 0.56, light color, F_2, 34_ = 10.77, *p* < 0.001, *η*^2^ = 0.20, effect on wakefulness, F_2, 34_ = 20.80, *p* < 0.001, *η*^2^ = 0.35, and effect on concentration, F_1.44, 24.55_ = 4.93, *p* = 0.02, *η*^2^ = 0.13. The error bars represent the standard deviation. The individual ratings are shaded.

Statistical analysis of visual comfort reveals a significant main effect of condition for all six dimensions of the questionnaire (see Figure A2 in Appendix B). Descriptively, both light exposures were rated as being too bright (M_small_ = 1.92; M_large_ = 2.22), slightly glaring (M_small_ = 3.65; M_large_ = 2.93), and unpleasant (M_small_ = 3.90; M_large_ = 3.52). In terms of brightness, the small solid angle was perceived as less pleasant compared to the large solid angle (t_16_ = 2.27, *p* = 0.037) and less glaring (t_16_ = 3.57, *p* = 0.002). The lighting in the dim light condition was considered to be more tiring (M_dim_ = 4.09) (t_17_ = 3.80, *p* = 0.001) and to impair concentration ability to a greater degree (*M*_dim_ = 3.72) (t_17_ = 2.36, *p* = 0.03) as opposed to the two lighting conditions (tiring: M_small/large_ = 2.66/2.57; impairing concentration: M_small/large_ = 3.06/2.77). There were no significant differences between the two different solid angles.

## 4. Discussion

We evaluated the effects of light exposure with two different solid angles compared to dim light on salivary melatonin concentration, subjective alertness, and cognitive performance in the evening. Consistent with the theoretical assumptions regarding circadian hormone variations, alertness modeling (e.g., [34]), and the occurrence of passive task-related fatigue (e.g., [35]), melatonin concentrations increased over time while subjective and objective performance decreased. Measurements of salivary melatonin confirmed the melatonin-suppressing effect of short-wavelength light (e.g., [53]). After 180 min, both lighting conditions attenuated melatonin by around 40% compared to a predicted suppression of 60% according to [54]. Obtained KSS ratings indicated an alertness-increasing effect of exposure to bright light. However, the present study does not provide evidence of an effect of bright light exposure with different solid angles on cognitive performance, as indicated by the reaction times in the PVT or miss rates in the MWCT. A possible reason may be that the MWCT results did not yield a sufficiently high power due to the small number of events. Nevertheless, this mismatch between effects on subjective and behavioral alertness measures is consistent with numerous studies that also demonstrated an influence of light exposure on subjective alertness but not on cognitive alertness in the PVT (e.g., [18,22,55,56,57,58]) or the MWCT (e.g., [59,60]). Since the general public is becoming increasingly aware of the alerting effects of light, expectations and placebo effects might have influenced the KSS ratings but not cognitive alertness [2].

In line with past research (e.g., [61,62]), it must be noted that both the magnitude and the onset of the evening melatonin increase varied greatly between participants. While a 180 min long light exposure of 40lx mEDI should be sufficient for an evening melatonin suppression of up to 50% [54], light intensities applied in this study were possibly not high enough to affect some individual participants (required light intensity can vary between 6 and 350lx [63]). This demonstrates the challenge of designing appropriate light applications for a wide range of people, as individuals have very different sensitivities to NIF light effects, which could be due to different genetic characteristics [64].

The present study further provides no evidence that the solid angle of light exposure affects the degree of melatonin suppression, subjective alertness, or cognitive performance during the evening. This is surprising because the light exposure with a large solid angle irradiates a larger retinal area in the eye and thus stimulates a larger number of ipRGCs. However, while the corneal illuminance remained constant in both light exposure conditions, light exposure with a small solid angle resulted in a higher retinal illuminance of the exposed portion of the retinal area.

In contrast, there are differences between the two light exposures regarding visual comfort. The light exposure with a large solid angle was rated more visually pleasant and less glaring. This can be explained by the fact that the perceived degree of visual comfort and glare of a lighting situation depends, among other things, on the luminance of the lighting surface and its contrast to the luminance of the background lighting within the visual field [65]. Since the luminance of the luminous surface was significantly higher in the small solid angle condition compared to the large solid angle, there was a stronger contrast to the dark background environment. These results corroborate recommendations for the design of lighting applications that consider NIF effects to favor larger solid angles for high corneal illuminances without visual discomfort [66].

Nevertheless, it should be taken into account that the sample used in this study consisted of a very homogeneous group of subjects and the results cannot be generalized for women and other age groups. Women are known to differ from men in the biological mechanisms involved in sleep regulation as well as sensitivity to the NIF effects of light [67,68,69]. Moreover, the experimental design reflects the real-world conditions only to a limited extent. Especially when driving at night, the driver’s eyes are exposed to more dynamic lighting conditions compared to the lab. Light produced by urban infrastructure or other traffic participants outside the vehicle can reach the interior, thus affecting the overall lighting conditions inside the car. The upright seated position with a defined eye position, as used in the laboratory setting, also does not correspond to a realistic seating position inside a car. To better understand the influence of the solid angle of light exposure, different solid angle sizes, brightness levels, and spectra should be further investigated. With regard to future applications in the field, it should also be mentioned that late-evening light exposure can induce circadian phase delays and hence cause adverse effects, such as reduced sleep quality at home and increased sleepiness on the following morning [70]. However, to date there is only limited knowledge available regarding the effects of evening light exposure, especially for shift work [71]. Additional empirical research, both under strictly controlled conditions and in the field, is necessary to design NIF-effective automotive interior lighting.

## 5. Conclusions

The aim of this study was to demonstrate the NIF effects of two light exposures with different solid angles in a controlled laboratory environment. A lower increase in salivary melatonin and a reduced decrease in subjective alertness was shown during the 3 h evening light exposure. While cognitive performance declined with progressing evening hours, subjects revealed no improvement in performance during light exposure. There was no difference in subjective alertness and salivary melatonin between the two solid angles. Thus, it can be summarized that a luminaire creating a solid angle of 0.05sr (at ~120lx mEDI) was sufficient to influence the subjective alertness as well as melatonin concentration in the evening. The light exposure with a large solid angle, however, was rated as visually more pleasant compared to that with the smaller solid angle. Thus, a trade-off must be made when designing appropriate lighting applications: while a small luminous area is associated with a lower cost of the device, a larger luminous area offers the option to produce higher corneal illuminances without causing visual discomfort.

## Data Availability

The datasets are available from the corresponding author on reasonable request.

## Appendix A

Appendix A contains material on study design, experimental setup and light setting.

**Table A1:** Illuminance, CCT, α-opic irradiances, melanopic efficacy of luminous radiation, melanopic daylight D65 efficancy ratio, and melanopic equivilant daylight D65 illuminance according to CIE S 026/E:2018 [48] in each experimental condition. Measured with the spectrometer UPRtek LED-Meter MK350S at the subjects’ eye position (cornea).

	Dim	Small Solid Angle	Large Solid Angle
photopic illuminance (lx)	1.06	101.96	98.94
CCT (K)	4982	12158	12146
S-cone-opic irradiance (mW/m^2^)	0.76	124.24	119.90
M-cone-opic irradiance (mW/m^2^)	1.44	155.80	151.40
L-cone-opic irradiance (mW/m^2^)	1.73	165.42	160.23
rhodopic irradiance (mW/m^2^)	1.31	169.95	164.78
melanopic irradiance (mW/m^2^)	1.16	162.22	156.80
mELR (melanopic efficacy of luminous radiation; mW/lm)	1.09	1.59	1.58
mDER (melanopic daylight D65 efficancy ratio)	0.82	1.20	1.19
mEDI (melanopic equivilant daylight D65 illuminance; lx)	0.87	122.32	118.23

## Appendix B

Appendix B contains material about the results of the study presented in the present paper.
